# Advances in enhanced mesenchymal stem cell technologies: innovations and therapeutic applications

**DOI:** 10.3389/fcell.2026.1868610

**Published:** 2026-07-20

**Authors:** Shanshan Zheng, Yukang Huang, Xianli Yang, Xun Mao, Juan Hong, Menglin Tang, Ping Yi, Yanmin Xu, Cheng Qian

**Affiliations:** 1 College of Life Sciences and Medicine, Zhejiang Sci-Tech University, Hangzhou, China; 2 IND Center, Chongqing Institute of Precision Medicine and Biotechnology Co., Ltd., Chongqing, China; 3 The Third Affiliated Hospital of CQMU (FangDa Hospital), Chongqing, China; 4 Department of Obstetrics and Gynecology, The First Affiliated Hospital of Chongqing Medical University, Chongqing, China; 5 IND Center, Chongqing Precision Biotech Co., Ltd., Chongqing, China; 6 Center for Precision Medicine of Cancer, Chongqing Key Laboratory of Translational Research for Cancer Metastasis and Individualized Treatment, Chongqing University Cancer Hospital, Chongqing, China

**Keywords:** mesenchymal stem cells, MSC enhancement, survival, homing efficiency, immunomodulatory efficacy, clinical trials

## Abstract

Mesenchymal stem cells (MSCs) have attracted considerable attention for clinical translation in regenerative medicine, primarily due to their validated paracrine effects, prominent immunomodulatory properties, and superior multipotent differentiation capabilities. However, the limited homing efficiency and poor post-transplant survival of MSCs severely compromise therapeutic efficacy, thereby giving rise to suboptimal and inconsistent treatment outcomes. To circumvent these critical drawbacks and fully harness the therapeutic potential of MSCs, researchers have incorporated a diverse array of effective strategies, including genetic engineering, preconditioning with cytokines, small molecular compounds or hypoxic stimuli, and scaffold-based culture systems. Given these promising research advances, this review systematically summarizes recent advances in MSC-enhanced therapeutic strategies, and elaborates on their core molecular mechanisms as well as how these mechanisms modulate MSC survival, homing capacity and immunomodulatory efficacy. On this basis, we further analyze the practical applicability of these enhanced MSCs in clinical trials and seek to provide critical insights for the clinical selection of MSC-based enhanced therapies.

## 1 Introduction

Mesenchymal stem cells (MSCs), a type of multipotent adult stem cells, are characterized by self-renewal and multi-lineage differentiation (Noronha et al., 2019). In recent decades, basic and clinical research into MSCs have experienced an exponential expansion, with burgeoning advances across multiple research directions (Han et al., 2025). Taken together, these studies have consistently delineated the unique biological characteristics of MSCs, which not only underpin but also their promising potential in regenerative medicine, immunomodulation, and cell-based therapy.

MSCs can be classified into diverse subtypes according to their tissue of origin, including bone marrow-derived MSCs (BMSCs), adipose tissue-derived MSCs (AD-MSCs), umbilical cord-derived MSCs (UC-MSCs), umbilical cord blood-derived MSCs (UCB-MSCs), and Wharton‘s jelly-derived MSCs (WJ-MSCs) and so on (Li et al., 2022). Notably, all MSC subtypes exhibit remarkable multilineage differentiation potential, which enables them to differentiate into osteoblasts, chondrocytes, adipocytes, myocytes, neurons, and stromal cells. Moreover, MSCs also exhibit potent paracrine effects, secreting bioactive cytokines and functional exosomes enriched with proteins and miRNAs (Nakao et al., 2021). By virtue of these secreted bioactive factors, MSCs mediate a spectrum of pivotal biological functions, encompassing immunomodulation, anti-apoptosis, tissue regeneration, and the promotion of angiogenesis. Additionally, MSCs display low immunogenicity due to their low MHC-I expression and the absence of MHC-II and critical costimulatory molecules, thus greatly favoring allogeneic transplantation (Keshavarz Shahbaz et al., 2022).

Taken together, MSCs possess a repertoire of prominent advantageous traits, including ample tissue origin, facile isolation protocols, efficient large-scale *in vitro* proliferative capacity, negligible immunogenicity and a favorable safety profile. As of April 2026, the WHO International Clinical Trials Registry Platform (ICTRP) shows that a total of 1,470 clinical trials targeting MSCs have been formally registered worldwide from multiple countries and regions including the United States, China, the European Union, and Japan. Furthermore, 19 therapies based on MSCs have received regulatory approval for the treatment of diseases.

Despite these notable advantages, MSCs translational potential is severely compromised by unsatisfactory outcomes in clinical trials and validation studies. Here, we systematically summarized the latest advances in MSC optimization strategies, analyze their functional mechanisms, and reviewed the current clinical application status of representative optimized approaches.

## 2 Strategies for MSC optimization

**FIGURE 1:**
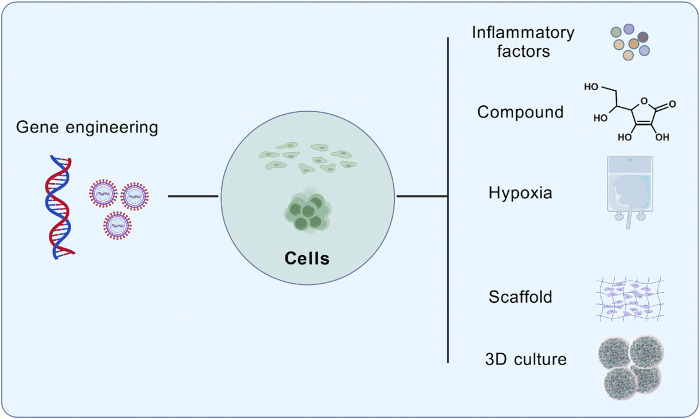
Strategies for MSCs optimization. To boost MSC therapy success, researchers have proposed strategies including genetic engineering and rational optimization of culture conditions. The latter includes inflammatory factors, compounds and hypoxic preconditioning, scaffold-based systems and 3D culture. The figure was prepared using BioGDP.com (Jiang et al., 2025).

MSCs have demonstrated extensive therapeutic potential in clinical applications, with prominent efficacy in immune modulation and tissue repair. Nevertheless, their clinical utility is hindered by two major limitations: 1) inadequate homing efficiency to pathological lesion sites after intravenous injection (Xu et al., 2022); and 2) compromised cellular viability and rapid apoptosis triggered by the hostile microenvironment, which is hallmarked by severe ischemia, hypoxia, inflammatory stress, and excessive oxidative stress (Wang et al., 2025). To surmount these aforementioned bottlenecks and further boost MSC homing capacity and survival rates, a diverse array of sophisticated strategies has been devised from different perspectives, including genetic engineering (Li D.-Y. et al., 2024), hypoxic preconditioning (Cao et al., 2025) cytokine pretreatment (Chang et al., 2021), and biomaterial scaffold-assisted cell delivery systems (Kamali et al., 2019) (Figure 1).

### 2.1 Genetic engineering of MSCs

With the rapid advancement of synthetic biology, the diversity of gene functions and the increasing sophistication of transgenic technologies have provide vast opportunities for the genetic engineering of MSCs. Ectopic overexpression of anti-apoptotic factors, including heat shock proteins (HSPs), bioactive cytokines and chemokines, can robustly promote MSC survival and proliferative potential *in vitro* and *in vivo*. Additionally, targeted gene knockout and RNA interference (RNAi) represent complementary strategies for the rational genetic engineering of MSCs.

#### 2.1.1 Cytokines and anti-apoptotic proteins

To tackle the challenge of poor survival rate MSCs in hostile microenvironment, researchers routinely adopt genetic engineering to enable MSCs to ectopically overexpress bioactive growth factors and anti-apoptotic genes (e.g., *HSPs* and *BCL-2*), which serves to robustly augment MSCs survival potential and intrinsic therapeutic efficacy. For example, in diseases including myocardial infarction (MI) and stroke, the injured areas are predominantly featured by severe ischemic-hypoxic condition (Cao Y. et al., 2022), which elicits a vigorous inflammatory and oxidative stress cascade that induces widespread cellular apoptosis (Yamagata, 2020; Yurista et al., 2023; Tian et al., 2025). Studies have shown that overexpression of *HSP70 *(Jin et al., 2020) can enhance the survival rate and anti-apoptotic ability of MSCs in acute lung injury (ALI) rats through the *PI3K/Akt* pathway. Analogously, hepatocyte growth factor (HGF) (Nie et al., 2026) have been demonstrated to activate the *Akt* signaling pathway concomitantly enhancing the paracrine secretion of MSCs, this cascade of effects robustly augments MSC viability and survival rates in the adverse pathological microenvironment. Similarly, vascular endothelial growth factor (VEGF) (Ni et al., 2017) and basic fibroblast growth factor (b-FGF) (Li K. et al., 2025) also improved the survival of MSCs in oxygen glucose deprivation (OGD) and MI *in vitro* models by potentiating their paracrine secretory capacity. Furthermore, fibroblast growth factor-21 (FGF-21) (Linares et al., 2020) effectively suppressed caspase activation, which in turn mitigates apoptotic cell death of MSCs exposed to H_2_O_2_. In addition, the direct ectopic overexpression of *BCL-2* (Ni et al., 2017) and *Akt*1 (Zhou et al., 2014) exerts a potent anti-apoptotic effect, protecting MSCs against apoptotic injury in OGD and Graft-versus-host disease (GVHD).

While these mentioned genes display distinct molecular regulatory mechanisms, they primarily target on the *Akt* signaling pathway, along with the modulation of BCL-2 and caspase family proteins. As a central signaling hub governing cell survival, Akt orchestrates the synergistic crosstalk of multiple downstream pathways in MSCs. It potently suppresses caspase activation, upregulates BCL-2 expression, and blocks mitochondrial-mediated apoptotic cascades, while concomitantly augmenting metabolic adaptive capacity and paracrine secretory function. Researchers can rationally choose wild-type *Akt* or hypoxia-responsive element (HRE)-driven *Akt* constructs for targeted genetic engineering of MSCs.

#### 2.1.2 Chemokine receptors and intercellular adhesion molecule

In addition to poor survival, insufficient homing efficiency constitutes an additional critical barrier that severely impedes the therapeutic applications of MSCs. To address this barrier, numerous studies have confirmed that the ectopic overexpression of chemokine receptors and adhesion molecules significantly enhances MSC homing capacity to pathological injury sites through the modulation of diverse signaling pathways. Table 1 shows the homing-promotion effects of chemotactic gene.

**TABLE 1:** Homing-promotion effects of chemotactic genes overexpression.

Gene	MSCs type	Signaling pathway	Indications	References
*CCR2*	UC-MSCs	*CCR2/CCL2*	Acute liver failure (ALF)	Xu et al. (2022)
*CXCR5*	MSCs	*CXCR5/CXCL13*	Cecal ligation and puncture	Shi et al. (2025)
*CXCR2*	MSCs	*IL-8-CXCR1/2*	Lung cancer	Yang et al. (2023)
*CCR1*	MSCs	*CCR1/CCL7*	Stress urinary incontinence	Jiang et al. (2020)
*CXCR4*	MSCs	*NLRP3/ASC/GSDMD*	Cardiac arrest-induced brain injury	Liu et al. (2025)
*ICAM-1*	MSCs	*—*	IBD	Li et al. (2019)
			GVHD	Tang et al. (2018)
*EP2*	MSCs	*EP2/PGE2*	ARDS	Han J. et al. (2016)
*CXCR7*	UC-MSCs	*—*	Lung fibrosis	Xiao et al. (2023)

Adapt from direct overexpression, some non-coding RNA, such as microRNAs have been reported to regulate the endogenic chemokine receptor/ligand. For example, downregulating *miR-141* (targeting *ICAM-1*) and *miR-139* (targeting *CXCR4*) (Zhao et al., 2021) have been shown to enhance MSC homing in ulcerative colitis mice.

**FIGURE 2:**
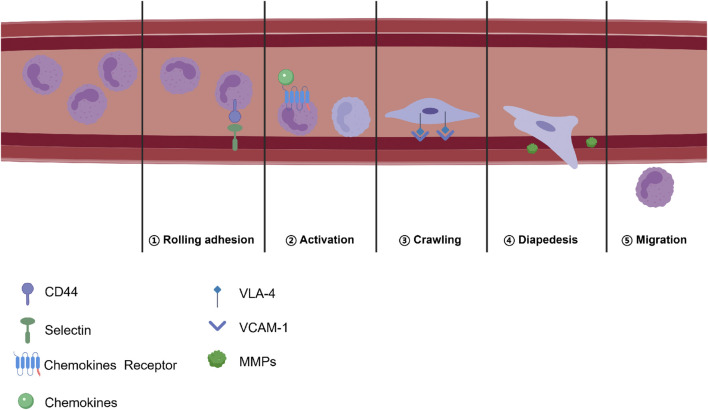
Homing cascade of MSC. Circulating MSCs were first captured by the vascular wall and formed a low-affinity contact. Then, they slowed down and eventually stopped rolling. Through adhesion molecules, they achieved firm attachment and crawling on the vascular wall. Then, the cells polarized and sought for the endothelial exit. Finally, they passed through the endothelial, basement membrane and pericyte barriers, and migrated towards the interstitial space guided by chemokines. The figure was prepared using BioGDP.com (Jiang et al., 2025).

Although, chemokine play an important role during MSC homing, it not the sole decisive factor. Homing cascade of MSCs could be roughly divided into five stages (Figure 2). At the first stage, the CD44 molecules on the surface of MSCs interacted with the E-selectin or P-selectin on the surface of endothelial cells, causing the MSCs to “roll” along the vascular wall (Sackstein et al., 2008). Then, the inflammatory factors (signaling molecules) released by the damaged tissues would activate the chemokine receptors on the surface of MSCs. After activation, the structure of the integrin molecules on the surface of MSCs changes, and their adhesion ability significantly increases. Different chemokine receptors, such as CXCR4 which binds to the inflammatory signaling molecule SDF-1, always play important roles at this key stage. The activated integrins tightly bind to the adhesion molecules on the endothelial cells (Ghadge et al., 2011). The most important pairing is the integrin VLA-4 on the surface of MSCs with the VCAM-1 on the surface of the endothelial cells (Brooke et al., 2008). After crawling, MSCs secreted matrix MMPs (metalloproteinases), particularly MMP-2, to degrade the basement membrane beneath the vascular endothelium and create pathways to the tissues. Subsequently, the MSCs deformed and passed through the intercellular spaces of the endothelial cells, completing the migration from within the blood vessel to outside the vessel. Within the tissue, the chemotaxis and homing regulation of MSCs was more complex because of the combined effect of the physical and the chemical microenvironment. Overexpression of designed chemokine receptors could promote the MSCs migrate to the lesion (Nitzsche et al., 2017).

In practical application, rational selection of candidate genes to potentiate MSC homing capacity necessitates precise matching of gene functional properties to the distinct pathogenic mechanisms and location of target lesions. Specifically, in MI, the infarcted myocardium exhibits robust SDF-1 expression (Zhao et al., 2022), making *CXCR4* the premier candidate gene for targeted genetic modification of MSC. In ALF, constitutive hepatic overexpression of CCL2 (Xu et al., 2022) designates *CCR2* as the optimal choice for MSC engineering. Similarly, *CXCR5* (a specific receptor for CXCL13) represents the optimal candidate gene for MSC modification, which is characterized by CXCL13 upregulation at lesion sites in chronic heart failure (Surówka et al., 2025).

### 2.2 Optimization of MSC culture process

Although genetic modification could markedly and stably enhance MSCs functionality, it also raises safety concerns, such as genotoxic risk from genetic modification, unintended off-target effects. Therefore, researchers have attempted to achieve the desired phenotype or high expression of the target gene in MSCs by altering the culture conditions. Based on the adaptive microenvironments for MSCs, pretreatment with hypoxia (Lee and Kang, 2020), bio-activator (Bui et al., 2021), scaffolds (Noronha et al., 2019), or 3D culture (Noronha et al., 2019) can significantly enhance their therapeutic potential.

#### 2.2.1 Cytokines pretreatment

Among various approaches, pretreatment with cytokines represents one of the most extensively utilized strategies to enhance the survival, homing and therapeutic efficacy of MSCs. Studies have shown that pretreatment with cytokines enhances the therapeutic efficacy of MSCs in multiple diseases, such as colitis (Faghih et al., 2024), MI (Esmaeili et al., 2021; Wang et al., 2022; Najafipour et al., 2024), etc.

The survival capacity is the fundamental premise for MSCs to exert subsequent biological functions, which is tightly regulated by a variety of signaling pathways and effector molecules. Among them, the key mechanism is that relevant factors activate signaling pathways to upregulate BCL-2. For instance, TNF-α (Bai et al., 2017), Mitsugumin53 (MG53) (Ma et al., 2020), and Vaspin (Zhu et al., 2019) can upregulate BCL-2 through different signaling pathways, thereby promoting the survival of MSCs: TNF-α triggers the *NF-κB* pathway activation, MG53 regulates the *NLRP3/caspase-1/IL-1β* pathway, and Vaspin modulates the *MAPK/p38* pathway cascade. Furthermore, LPS (Wang et al., 2009), Erythropoietin (EPO) (Zhou S. et al., 2020), and b-FGF (Xu et al., 2016) have been demonstrated to possess this pro-survival function through *TLR4/PI3K/Akt, SIRT1,* and Kruppel-like factor 4 (*KLF4*) pathways, respectively. Additionally, SDF-1α (Esmaeili et al., 2021), low-concentration TGF-β1 (Li et al., 2016) also promote MSC survival.

Beyond their survival capacity, the homing potential of MSCs is closely correlated with the expression of the CXCR4. Notably, TNF-α (Bai et al., 2017), SDF-1 (Nam et al., 2020), and EPO (Li K. et al., 2024) are not only crucial pro-survival factors but also key regulators that boost MSC homing by upregulating CXCR4 expression. Specially, TNF-α exerts this effect through the *NF-κB* pathway and EPO facilitates MSC homing by inhibiting the *Notch1/Jagged* pathway. In parallel, a variety of other factors—including TGF-β1 (Dubon et al., 2018), LPS (Kim et al., 2016), MG53 (Ma et al., 2020; 2022), platelet lysate (PL) (Yan et al., 2020), IFN-γ (Shao et al., 2025), IL-1β (Nie et al., 2020), and b-FGF (Lu et al., 2023) further strengthen MSC homing through diverse mechanisms, which involve the non-canonical signaling/N-cadherin axis, as well as *TLR4/IRF1/NF-κB/PI3K*, *Nrf2*, and *AMPK/mTOR* pathways.

Notably, the regulatory effects of some factors extend far beyond merely enhancing MSC survival and homing, as they can also elicit the robust immunomodulatory properties of MSCs. Previous studies have shown that MSCs exert immunomodulatory effects through multiple pathways. For example, upon stimulation with IFN-γ, MSCs express indoleamine 2,3-dioxygenase (IDO), which catalyzes the conversion of tryptophan to kynurenine, leading to local tryptophan depletion and kynurenine accumulation in the coculture supernatants; both of these events inhibit allogeneic T-cell proliferation in mixed lymphocyte reactions, as evidenced by the finding that the addition of tryptophan significantly restores T-cell proliferation (Meisel et al., 2004). In addition, MSCs suppress T-lymphocyte proliferation through the production of soluble factors. Using neutralizing monoclonal antibodies, TGF-β1 and HGF have been identified as the key soluble mediators of this suppressive effect (Di Nicola et al., 2002). MSCs also suppress T cell proliferation via the engagement of the inhibitory molecule programmed death-1 (PD-1) with its ligands PD-L1 and PD-L2 (Augello et al., 2005). Moreover, MSCs secrete IL-10 to induce the generation of regulatory dendritic cells (Liu et al., 2012) and macrophages (Nie et al., 2024). Furthermore, MSC-derived exosomal miRNAs exert immunomodulatory effects by regulating T cells, dendritic cells, and macrophages (Shahsavandi et al., 2025). Building upon these signaling pathways, cytokine preconditioning further amplifies the immunomodulatory capacity of MSCs by strategically leveraging the same molecular machinery.

For instance, TNF-α and LPS-preconditioned MSC-derived exosomes enhance MSC immunomodulatory ability by promoting induction of anti-inflammatory M2 macrophage polarization (Nakao et al., 2021). LPS act through *miR-150–5p*/*PI3K/Akt/mTOR* (Zheng et al., 2024) and *NF-κB/NLRP3/*procaspase-1/*IL-1β* (Zhang et al., 2023) signaling pathways. LPS stimulation can also induce the expression of IDO in MSCs in a TLR4-dependent manner to enhance the immunosuppressive effect of MSCs (Waterman et al., 2010). Moreover, MSC pretreated with IFN-γ, TNF-α and IL-1β could upregulate the expression of PD-1 ligands (PD-L1 and PD-L2) and enhance the immunosuppressive effect on activated T cells (Hackel et al., 2023). In addition, MSCs pretreated with IL-1β alone can improve inflammatory response and metabolism by secreting higher levels of IL-10 and TGF-β (Wang et al., 2023). Meanwhile, MG53 (Ma et al., 2020) protects hUC-MSCs from inflammatory damage by inhibiting the *NLRP3/Caspase-1/IL-1β* axis. It also works synergistically to enhance their ability to alleviate LPS-induced neuroinflammation.

**FIGURE 3:**
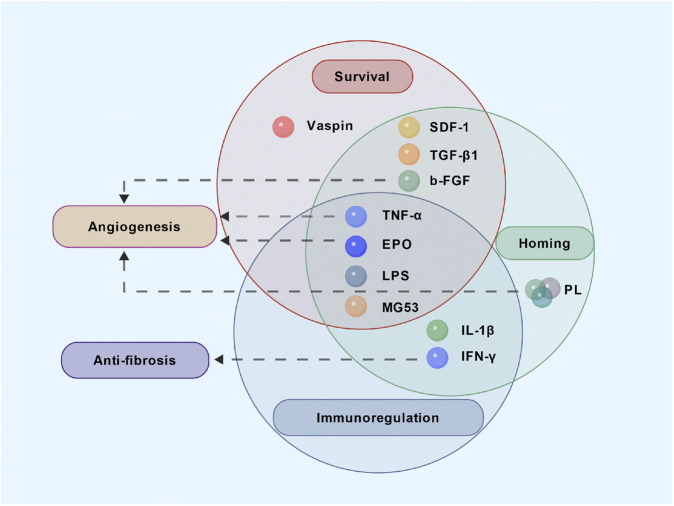
Core mechanism network of cytokine pretreatment strategies regulating MSC functions. Pretreatment of MSCs with different cytokines yields distinct enhancing effects. Most cytokine pretreatments augment multiple functional properties of MSCs: for instance, (1) pretreatment with TNF-α not only improves MSC survival and homing but also enhances their immunomodulatory activity and pro-angiogenic function. (2) pretreatment with SDF-1 and TGF-β1 promotes MSC survival and homing. (3) and IL-1β pretreatment facilitates MSC homing while reinforcing immunomodulatory capacity. Only a limited number of cytokine pretreatments exert a single enhancing effect-for example, Vaspin pretreatment solely enhances MSC survival. The figure was prepared using BioGDP.com (Jiang et al., 2025).

Furthermore, beyond the three core properties of survival, homing, and immunomodulation, these regulatory factors can also modulate additional functional characteristics of MSCs to expand their therapeutic potential. Specifically, b-FGF (Manshori et al., 2022), TNF-α (Cavallero et al., 2023), EPO (Iso et al., 2023), and PL (Zhang et al., 2025) can augment the angiogenic capacity of MSCs, while IFN-γ (Kanai et al., 2021) is able to promote their anti-fibrotic activity (Figure 3).

In summary, the utilization of pleiotropic core factors (e.g., TNF-α, LPS, and MG53) for pretreatment simultaneously enhances the three critical functions of MSC-survival, homing, and immunomodulation, thereby more comprehensively and robustly improving MSC functional properties. However, cytokine-based preconditioning also faces inherent challenges, including the difficulty in determining optimal treatment conditions and the high costs incurred in large-scale production. These limitations could potentially be mitigated by screening for cost-effective alternatives, such as functionally similar cytokines or small molecule compounds.

#### 2.2.2 Compounds pretreatment

To address the aforementioned challenges of cytokine preconditioning in clinical translation, bioactive compounds have emerged as promising and effective alternatives. These compounds including natural extracts and synthetic molecules, can modulate MSC functions through molecular pathways analogous to those of cytokines. Notably, they possess prominent advantages of lower production costs and superior physicochemical stability, making them much more suitable for clinical translation applications.

A variety of natural compounds can protect MSCs from oxidative stress injury and apoptosis through three perspectives: (1) activation of the classic cell survival signaling pathways; (2) apoptosis-related gene expression; (3) antioxidant enzyme activity and reactive oxygen species (ROS) regulation. These natural compounds and their function mechanism were listed in Table 2. It was obvious that many of the mechanisms of action are related to *PI3K/Akt*. *PI3K/Akt* is the core signaling hub for cell anti-apoptosis and antioxidant stress response. Multiple endogenous protective signals converge here. Natural compounds often have multi-target characteristics and can indirectly activate this pathway by acting on upstream receptor tyrosine kinases, G protein-coupled receptors, or inhibiting negative regulatory factor *PTEN*, etc. Moreover, in the H_2_O_2_-induced oxidative stress model, the cells dependence on *PI3K/Akt* increases. Some compounds may indirectly restore or enhance the stress-induced activation of this pathway by eliminating reactive oxygen species (ROS), thereby exhibiting an “activated” phenotype. In summary, although the central role of *PI3K/Akt* was clear, the upstream pathway and downstream effects of the *PI3K/Akt* involved in natural compounds were complex.

**TABLE 2:** Molecular mechanism of natural compound pretreatment on MSCs against apoptosis.

Stimuli	MSCs type	Signaling pathway	Indications	References
Oleoylethanolamide	AD-MSCs	*Nrf2/NQ O -1/H O -1*	—	Zare et al. (2023)
Astaxanthin				Mohammadi et al. (2021)
Neochlorogenic acid	BMSCs		Intervertebral disk degeneration	Fang et al. (2024)
Chrysin		*PI3K/Akt/Nrf2*	Type 1 diabetes	Li and Wang (2022)
High-density lipoprotein		*PI3K/Akt*	MI	Xu et al. (2012)
Selenomethionine		*PTEN/PI3K/Akt*	Osteointegration	Li Y. et al. (2021)
Vitamin C/E/D3	WJ-MSC	*IGF-1/PI3K/Akt*	—	Ashfaq et al. (2020)
Icaritin	UC-MSCs	*HGF/c-Met*	ALF	Wang et al. (2019)
Ginkgo biloba L. extract	BMSCs	*p38/MAPK/JNK*	—	Wang A. et al. (2018)
Sex hormone-binding globulin	AD-MSCs	*ISR-1/PI3K/Akt/GLUT4*	Equine metabolic syndrome	Bourebaba et al. (2023)
Staphylococcal enterotoxin B	MSCs	*Bcl2*↑, *Bax, P53, and p21*↓	Sepsis	Saeedi et al. (2019)
Cladophora glomerata methanolic extract	AD-MSCs	*Bcl2*↑, *p21, p53, Bax and Casp-9*↓, SOD and Catalase↑, ROS↓	Metabolic syndrome	Bourebaba et al. (2019)

Actually, some natural compounds also have other additional functions as well. For example, pretreatment with natural compounds also promotes MSC homing through the direct or indirect modulation of CXCR4 expression. Rapamycin (Zheng et al., 2019), Deferoxamine (Isildar et al., 2025), Heparin (Suzuka et al., 2022), and GSK-3β inhibitor (Kim et al., 2013) have been reported to upregulate CXCR4 expression, thereby facilitating the homing of MSCs toward inflammatory and ischemic target sites.

The multiple functions of natural compounds have brought about extensive benefits for the functions of MSCs. However, the complexity and uncertainty of their function mechanisms also bring potential risks. Recent studies have demonstrated that certain clinically approved synthetic compounds serve as effective pretreatment agents for MSCs, with the capacity to markedly boost their therapeutic functions of promoting survival, enhance homing, regulate immunity. Compared to natural compounds, clinically approved compound may have a clearer mechanism and better safety.

For example, melatonin which has been approved for clinical application is an endogenous hormone secreted by the pineal gland in the brain (Yu et al., 2026). Melatonin pretreatment attenuates oxidative stress-induced MSC apoptosis by activating *PI3K/Akt* (Huang et al., 2023) pathway. Since MSCs exhibit poor homing capability toward disease targets, Sodium valproate (VPA) pretreatment can promote cell homing in ischemia/reperfusion injury (IRI) by activating *Akt/PI3K* and *SDF1/CXCR4* pathways (Shokeir et al., 2024). In addition to promoting MSC survival and homing, synthetic compounds can also enhance their immunomodulatory functions. Pretreatment with recombinant human growth differentiation factor 7 (rhGDF7) (Tao et al., 2023) activated the *AMPK* pathway and ameliorated inflammation, oxidative stress, and neural damage in the brains of mice with cerebral ischemia/reperfusion (I/R) injury. Table 3 summarizes synthetic compounds, MSCs sources and their mechanisms of action in multiple diseases.

**TABLE 3:** Synthetic compounds with enhancing effects.

Stimuli	MSCs type	Effects (signaling pathway)	Indications	References
Melatonin	Nucleus pulposus-derived MSCs	Alleviated MSCs apoptosis (*PI3K/Akt*)	Intervertebral disc degeneration disease	Huang et al. (2023)
	AD-MSCs	Increased survival and homing of MSCs	Osteoarthritis	Jiang et al. (2024)
		Increased MSC homing and differentiation	Spinal cord injury (SCI)	Naeimi et al. (2022)
Atorvastatin	MSCs	Improved MSC homing (*miR-146a/CXCR4*)	MI	Li N. et al. (2021)
Low-concentration H_2_O_2_	AD-MSCs	Increased MSC survival (*Nrf2/H O -1*)	—	Garrido-Pascual et al. (2020)
D-Alanine 2, Leucine 5 Enkephaline	MSCs	Increased MSC survival	Hypoxia-reperfusion	Mullick and Sen (2018)
5-azacytidine	MSCs	Enhanced MSCs homing (*COX2/PGE2*)	Immune disease	Lee et al. (2015)
rhGDF7	BMSCs	Averted inflammation and oxidative stress	I/R	Tao et al. (2023)
VPA	WJ-MSC	Enhanced MSCs homing (*Akt/PI3K, SDF1/CXCR4*)	IRI	Shokeir et al. (2024)
VCAM 1 antibody	MSCs	Enhanced MSC homing	Colitis	Chen et al. (2019)
All-trans-retinoic acid	WJ-MSC	Increased MSC survival	Renal ischemia	Barakat et al. (2022)
Ceramide-1-phosphate	MSCs	Improved MSC homing, proliferative, and anti-inflammatory activities (*MAPK/Akt*)	PAH	Lim et al. (2016)

In short, there are many compounds alternative, and in fact their underlying activation pathways are very similar. In practical applications, in addition to considering the benefits of compound pretreatment, we should pay more attention to the safety and process compatibility of the selected options.

#### 2.2.3 Hypoxia pretreatment

MSCs are naturally adapted to hypoxic microenvironments *in vivo* (Li et al., 2022)—this critical physiological trait that has inspired the development of hypoxia pretreatment strategies for MSCs. The cellular response to low oxygen tension is primarily orchestrated by hypoxia-inducible factor 1 (HIF-1). HIF-1 is a heterodimeric (composed of HIF-1α and HIF-1β) transcription factor that binds to hypoxia response elements. Under hypoxic conditions, the oxygen-dependent proteasomal degradation of the oxygen-sensitive subunit HIF-1α is inhibited, thereby promoting transcriptional activation and mediating various physiological and cellular mechanisms required for adaptation to hypoxia. By simulating physiological hypoxia to pretreat MSC, these cells can be pre-activated, which in turn mainly augments the survival and homing effect of MSCs (Zhuo et al., 2024). Hypoxia pretreatment usually employs oxygen concentration ranging from 0.1% to 2%, and varying oxygen levels in this range can elicit distinct functional effects on MSCs.

For instance, Sang Hun Lee’s team demonstrated that hypoxia preconditioning (2% oxygen) enhances MSC survival, proliferation, and angiogenic cytokine secretion in a murine hindlimb ischemia model. This enhancement occurred via activation of *HIF-1α/GRP78/Akt* (Lee et al., 2017) signaling axes, which in turn activated the *JAK2/STAT3* (Han Y.-S. et al., 2016). Consequently, the treatment led to improved functional recovery of the ischemic tissue. Additionally, the team led by Xinyang Hu from Zhejiang University College of Medicine discovered that BMSCs preconditioned with 0.5% O_2_ not only exhibited enhanced homing capacity (Hu et al., 2011), but also showed improved survival and angiogenic potential (Hu et al., 2008) in MI rats. The enhanced homing effect was mediated through the regulation of Kv2.1 in FAK phosphorylation/activation. Table 4 summarizes varying oxygen levels, MSCs sources and their mechanisms of action.

**TABLE 4:** Hypoxia pretreatment of MSCs.

Stimuli	MSCs type	Effects (signaling pathway)	Indications	References
2% O_2_	Embryonic stem cell-derived MSCs	Increased MSC viability	—	Lee et al. (2021)
	MSCs	Enhanced MSC survival and homing (*HIF-1α-GRP78-Akt/JAK2/STAT3*)	Hind-limb ischemia	Lee et al. (2017) Han Y.-S. et al. (2016)
1% O_2_	MSCs	Improved MSC survival and homing (*DANCR/miR-656-3p/HIF-1α*)	Preeclampsia	Haoran et al. (2023)
		Enhanced MSCs immunomodulation	—	Gupta et al. (2022)
	Amniotic MSCs	Improved MSC proliferation, homing, anti-apoptosis (*HGF/c-Met*)		Wang et al. (2024)
0.5% O_2_	BMSCs	Improved MSC survival and angiogenesis	MI	Hu et al. (2008)
		Improved MSC homing (*FAK*)		Hu et al. (2011)
0.1% O_2_		Attenuated MSC apoptosis and oxidative stress		Song Y. et al. (2022)

In conclusion, hypoxic preconditioning endows MSCs with more favorable biological characteristics and superior therapeutic potential. However, this promising strategy is confronted with several challenges: differing oxygen concentrations exert variable effects on MSC behavior, precisely controlled hypoxic conditions maintenance remains technically hurdles, and long-term efficacy and safety data are insufficient. For optimal therapeutic outcomes, future studies should be precisely tailored according to target tissue O_2_ levels and disease progression stage, with thoroughly assessing of both therapeutic efficacy and safety profiles.

#### 2.2.4 Scaffold-based systems and 3D culture

Compared with traditional two-dimensional (2D) cultivation, cells are actually in a complex three-dimensional (3D) microenvironment *in vivo*. This environment not only contains biochemical signals but also involves physical and topological structural cues. Both biomaterial scaffolds and 3D spheroids can faithfully reconstruct the native *in vivo* microenvironment, thus generating a permissive niche to govern the biological behaviors of MSCs, including activity, proliferation, and differentiation of MSCs.

##### 2.2.4.1 Scaffold-based systems

Scaffolds can recapitulate the native extracellular matrix (ECM) and construct an *in vivo*-like microenvironment for MSCs, while exhibiting favorable biocompatibility and tunable biodegradability. Scaffolds employed for MSCs culture are generally classified into three categories: 2D scaffolds, 3D static scaffolds, and 3D dynamic suspension scaffolds.

2D scaffolds, represented by chitosan membranes (Yeh et al., 2014), provide a suitable interface for MSCs adhesion and proliferation. 3D static scaffolds, such as poly lactic-co-glycolic acid (PLGA) (Zhao et al., 2019), hydrogels (Shen et al., 2020), acellular matrix (Yan et al., 2023), and 3D-printed scaffolds (Prasopthum et al., 2019), effectively mimic the ECM niche *in vivo*. In contrast, 3D dynamic suspension scaffolds, typified by microcarriers, afford a large specific surface area to support efficient cell expansion.

Shan-hui Hsu’s research team (Huang et al., 2011) indicated that chitosan-hyaluronic acid membranes can induce the formation of 3D spheroids through surface self-assembly. These spheroids maintained the expression of stemness marker genes in both adipose-derived and placenta-derived MSCs, while enhancing their chondrogenic differentiation potential. Mechanistically, the Rho/Rho-associated kinase (*ROCK*) signaling pathway may participate in regulating this spheroid formation process. In addition, Guo and Liu’s team found that exosomes derived from MSCs cultured on 3D porous acellular cartilage ECM (ACECM) scaffolds promoted the proliferation, homing, and chondrogenic differentiation of BMSCs. These exosomes also suppressed chondrocyte apoptosis under inflammation conditions and drove M2 polarization of macrophage through the *miR-125a/miR-29a/NF-κB p65/NLRP3* axis, ultimately contributing to the repair of osteochondral defects in rat knee joins (Yan et al., 2023).

In the field of microcarriers, Prof. Yanan Du’s team at Tsinghua University first reported their original biodegradable gelatin microcryogels (GMs) in 2014 (Li et al., 2014). The injectable 3D microscale cellular niches formed by GMs combined with AD-MSCs effectively improved the retention and survival of MSCs in a mouse model of critical limb ischemia (CLI). Building on this platform, the team further developed a scalable, automated, and closed “cell factory” for MSCs production. Notably, this technology supported the approval of China’s first stem cell drug-“Amimatoside Injection” (https://www.nmpa.gov.cn/), which is indicated for the treatment of acute GVHD in patients aged 14 years and older. A summary of scaffold-based culture systems for MSCs is provided in Supplementary Table S1. The data summarized in Supplementary Table S1 are derived from multiple studies (Yeh et al., 2014; Das et al., 2019; Prasopthum et al., 2019; Huang et al., 2021; Kuttappan et al., 2018; Kamali et al., 2019; Qiu et al., 2024; Zhou et al., 2019; Shen et al., 2020; Zhou C. et al., 2020; Beiki et al., 2018; Pan et al., 2024; Qi et al., 2021; Birhanu et al., 2018; Abdollahi et al., 2024; Su et al., 2017; Cui et al., 2020; Zhang et al., 2018; Wang B. et al., 2018; Casagrande et al., 2018; Rostami et al., 2020; Norouz et al., 2019; Regmi et al., 2021; Li D. et al., 2024; Song H. et al., 2025; Zeng et al., 2015; Kaur et al., 2024; Chen et al., 2025; Naseri Mobaraki et al., 2022; Zhou et al., 2023; Shekaran et al., 2016; Doron et al., 2023).

Scaffold-based culture systems provide mechanical support and modulate the cellular microenvironment, thereby protecting MSCs and enhancing their therapeutic functions. 2D scaffolds are easy to fabricate, cost-effective, and highly standardized, yet their scalability is restricted, rendering them suitable primarily for basic mechanistic studies. 3D static scaffolds established a customized microenvironment for MSCs, making them suitable for tissue engineering and regenerative implantation. However, they commonly exhibit diffusion limitations, which may cause central cell necrosis or functional heterogeneity. By comparison, 3D dynamic scaffolds yield superior mass transfer efficiency and supply appropriate physiological shear stress, enabling the large-scale, homogeneous, and high-viability cell expansion. This platform has become the preferred manufacturing strategy for clinical-grade cell products.

##### 2.2.4.2 3D spheroid culture

Unlike the scaffold-based 3D culture methods mentioned above, cell spheroids represent a scaffold-free 3D cell culture strategy. They effectively recapitulate the *in vivo* physiological microenvironment and strengthen cell–cell interactions, thereby significantly enhancing the biological functions and therapeutic properties of MSCs. To date, several well-established techniques have been widely used to generate 3D MSC spheroids. For example, the Hanging Drop technique (Lee et al., 2020) promotes cell aggregation through gravitational sedimentation of cell droplets. Magnetic-based Levitation (Chan et al., 2021), enables the assembly of magnetic nanoparticle-labeled cells under an external magnetic field. Low-Adhesion Culture (Raik et al., 2023) uses non-adherent substrates to inhibit cell attachment and drive spontaneous cell self-assembly. Meanwhile, rotary cell culture (Zhang et al., 2010) maintains cells in suspension via bioreactor-derived physical forces, thereby facilitating the formation of compact spheroids.

For instance, Researchers (Lee et al., 2020) observed elevated SOD2 expression in 3D MSC spheroids generated via the hanging drop method. This upregulation alleviated oxidative stress and apoptosis in MSCs, while promoting cartilage regeneration through modulation of the *PI3K/pAkt/pNrf2* and *pERK/pNrf2* signaling pathways. Furthermore, compared to monolayer MSC culture, MSC spheroids prepared by hanging drop culture also showed enhanced anti-inflammatory (Lee et al., 2022), Chemotaxis (Lee H.-T. et al., 2025), angiogenic activity (Deng et al., 2021). Rotary culture systems range from small-scale research platform-including shakers, oscillators, and spinner flasks-to large-scale bioreactors such as stirred-tank, rotating-wall, and perfusion systems, enabling high-efficiency cell expansion. Using a shaking-based 3D culture system, Niibe et al. (2020) generated MSC spheroids with augmented pluripotency. When combined with neurosphere culture, this approach yielded MSC spheroids with improved cell survival, immunomodulatory capacity and differentiation potential, ultimately enhancing *in vivo* bone regeneration (Ohori-Morita et al., 2022). Min Lim et al. (2023) developed an advanced 3D dynamic culture strategy using an optimized orbital shaking system supplemented with exogenous TGF-β3. This approach significantly increased exosome secretion from 3D WJ-MSC spheroids and strengthened their regenerative and immunomodulatory functions, thereby boosting therapeutic efficacy in a full-thickness excision wound model. In addition, Zhang ZY et al. (Zhang et al., 2010) reported that a biaxial rotating bioreactor greatly promoted cell proliferation in human fetal mesenchymal stem cells (hfMSCs), improved cellular spatial distribution, and enhanced osteogenic induction. The resulting tissue-engineered bone grafts effectively repaired critical-sized femoral defects in rats. Information on other methods, such as magnetic levitation culture and low-adhesion culture, is presented in Table 5.

**TABLE 5:** Types of 3D MSC spheroid culture.

Types	MSCs type	Enhanced effects (signaling pathway)	Indications	References
Hanging-drop 3D spheroid	UCB-MSCs	Anti-apoptosis, cartilage regeneration (*PI3K/pAkt/pERK/pNrf2*)	Osteoarthritis	Lee et al. (2020)
	MSCs	Angiogenesis and inflammation regulation	Spinal cord injury	Deng et al. (2021)
		Anti-inflammatory	Rheumatoid arthritis	Lee et al. (2022)
		Chemotaxis	—	Lee H.-T. et al. (2025)
Magnetic -based levitation	Dental pulp-MSCs (DPMSCs)	Trilineage differentiation capacities, anti-apoptosis (*MAPK/NF-kB*)	—	Chan et al. (2021)
Low-Adhesion Culture	DPMSCs	Stemness, differentiation, and regenerative abilities	Calvarial defect	Raik et al. (2023)
	UC-MSCs	Immunoregulation, stemness	Ovarian failure	Zhang Y. et al. (2024)
	AD-MSCs	Immunoregulation	Inflammatory diseases	Lee E. et al. (2025)
Rotary Culture	BMSCs	Survival, immunomodulation, differentiation, and bone regeneration	Bone regeneration	Ohori-Morita et al. (2022)
	WJ-MSCs	Paracrine, regeneration and immune regulation	Wound healing	Min Lim et al. (2023)

In summary, the hanging drop culture method is characterized by simple operation and low-cost, yet it is difficulty to precisely control the culture environment, often resulting in inconsistent spheroid sizes. Magnetic levitation culture enables rapid spheroid formation and facilitates convenient real-time observation; however, cell magnetization may interfere with subsequent quantitative analyses. Low-adhesion culture is suitable for high-throughput screening and facilitates easy collection and analysis, yet long-term culture remains challenging. As spheroids increase in size, interior cells may undergo necrosis due to insufficient nutrient and oxygen diffusion. By contrast, dynamic culture generates highly uniform and reproducible spheroids and supports large-scale production, but it is associated with high equipment costs and technical barriers. For basic research and investigations into cellular mechanisms, the hanging drop method is a favorable choice given its low cost and high flexibility. For large-scale cell manufacturing aimed at clinical translation, 3D microcarriers integrated with bioreactor systems are strongly recommended. These systems combine high throughput, standardization, safety, and efficacy, representing the mainstream strategy for industrial-scale cell production.

## 3 Clinical progresses

**FIGURE 4:**
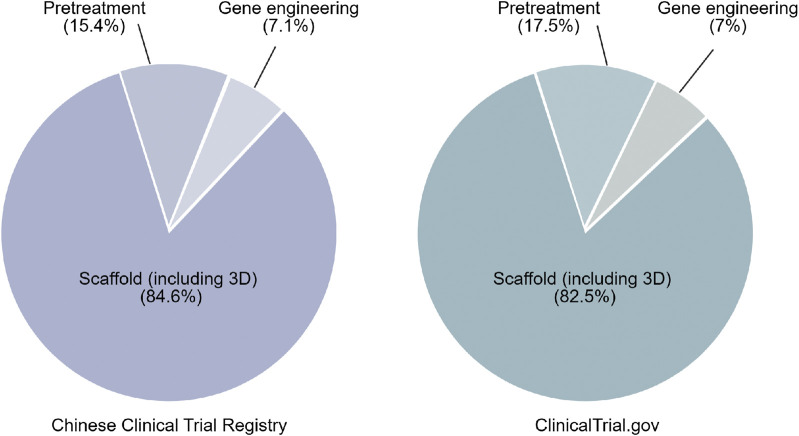
Proportion of different strategies in clinical trials. Among the clinical registration studies of enhanced MSCs recorded in the Chinese Clinical Trial Registry and ClinicalTrials.gov, scaffold-based MSC therapies (including 3D culture approaches) account for more than 80% in both databases. Pretreatment strategies other than scaffold-based methods each constitute over 15%, whereas genetic modification accounts for only approximately 7%. The figure was prepared using BioGDP.com (Jiang et al., 2025).

While there are 1,470 clinical trials and 19 approved products for unmodified MSCs, the number of clinical trials for enhanced MSCs remains strikingly lower—a stark contrast to their unmodified counterparts. We have compiled data from the Chinese Clinical Trial Registry and ClinicalTrails.gov regarding enhanced MSC therapies. The results show that, according to registry data updated as of April 2026, 18 clinical trials of functionally enhanced MSCs have been registered in the Chinese Clinical Trial Registry, and 50 trials are listed on ClinicalTrails.gov. Most of these trials focus on scaffold-based MSC therapies (including 3D culture) (>82%), with only a limited number investigating gene engineering, hypoxic preconditioning, and other pretreatment strategies Figure 4. Detailed characteristics of representative trials are summarized in Table 6.

**TABLE 6:** Clinical Trials of enhanced MSCs.

Strategies	Intervention	MSC Type	R and D institutions	Indications	Number	Phase	Start	Status
Scaffold	Sodium hyaluronate gel	UC-MSCs	Obstetrics and Gynecology Hospital of Fudan University	Endometrium damage	ChiCTR24000 80588	Phase I	Feb, 2024	Not yet recruiting
	Nanofat scaffold	AD-MSCs	The Third Affiliated Hospital of Air Force Medical University	Temporomandibula r Disorders	ChiCTR23000 69677		Mar, 2023	
Genetic engineering	Numb overexpression	BMSCs	Shuguang Hospital Affiliated to Shanghai University of Traditional Chinese Medicine	Compensatory cirrhosis	ChiCTR20000 38467	Exploratory study	Sep, 2020	
Hypoxia pretreatment	HIF-1α pretreating	MSCs	Shanghai Sixth People's Hospital	Nail bed defect	ChiCTR20000 30439		Mar, 2020	Recruiting
Scaffold	Chitosan Scaffold	AD-MSCs	Assiut University	Diabetic foot	NCT03259217	Phase I	Oct, 2017	Not yet recruiting
	Collagen I scaffold	BMSCs	Royan Institute	Knee Osteoarthritis	NCT00850187		Aug, 2008	Completed
	Neuro-Regen Scaffold™	MSCs	Chinese Academy of Sciences	Spinal Cord Injury	NCT02688049	Phase Ⅰ /Ⅱ	Jan, 2016	Enrolling by invitation
	Plasma fibrin hydrogel	AD-MSCs	A.A. Partners, LLC	Burns	NCT03113747		Mar, 2015	Recruiting
	Fibrin Matrix	Peripheral Blood MSCs	KLE Society's Institute of Dental Sciences	Dental Implants	NCT03044119	Phase I	Mar, 2018	
	Gel	UC-MSCs	Chinese PLA General Hospital	Skin Ulcers	NCT02685722		Jan, 2012	Completed
Genetic engineering	TRAIL overexpression	MSCs	University College, London	Adenocarcinoma of Lung	NCT03298763	Phase Ⅰ /Ⅱ	Mar, 2019	Recruiting
Hypoxia pretreatment	Hypoxic condition	AD-MSCs	Gadjah Mada University	Ligament Rupture	NCT04889963		Jan, 2021	

Scaffold-based MSC therapies are widely preferred, owing to the scaffolds’ favorable safety and biocompatibility. They enable cell adhesion, boost MSC differentiation and tissue regeneration, and have proven effective in clinical studies (Table 7). However, clinical evidence reveals that scaffold-based MSC therapies are predominantly in early-stage clinical trials (Phase I or I/II). We posit that the slow clinical translation progress may be due to a lack of standardized GMP (Good Manufacturing Practices) protocols (Williams et al., 2025), variable biological responses of scaffold-MSC constructs to human microenvironments (Lopes-Pacheco and Rocco, 2023), and insufficient long-term safety evidence (Cao C. et al., 2022). Nevertheless, scaffold-based approaches will remain a key research focus in the near future.

**TABLE 7:** Advantages of scaffold-MSCs.

Assessment criteria	Scaffold-based MSCs	Other methods
**Technology Maturity**	Well-established research foundation, possessed safety, biocompatibility and functionality (★★★★★)	Complex *ex vivo* manipulation, potential abnormal cellular functions, genomic instability or immune rejection risks (★★☆☆☆)
**Regulatory adaptability**	Classified as a medical device, well-established approval process (★★★★☆)	Classified as ATMPs, enhanced preclinical data requirements and long-term follow-up studies (★★★☆☆)
**Ethics arguments**	“Nature-Based” therapeutic augmentation approach (★☆☆☆☆)	Concerns over “excessive artificial intervention” arising from genetic modifications (★★★☆☆)
**Clinical evidence**	Clinical therapeutic outcomes substantiated by multicentric trials, validated paths preferentially selected by researchers (★★★★☆)	Preclinical potential in animal models, translational hurdles in clinic (★★☆☆☆)

## 4 Discussion

This article systematically reviewed various strategies to improve the clinical efficacy of MSCs, such as gene modification and optimization of culture conditions. Although these methods have shown potential therapeutic potential in preclinical and clinical studies, the current MSCs modification regimen still faces certain limitations. Therefore, further research is urgently needed to address these challenges (Table 8).

**TABLE 8:** Limitations and solutions of enhanced MSCs.

Limitations	Solutions
Potential tumorigenicity of modified cells	(1) Safer methods such as CRISPR-Cas9 site-specific insertion and RNA-based non-integrating systems (2) Integration site assessment, clone formation assayetc.
Toxicity of high dose small molecule preconditioning	(1) Choose approved medicine (2) Washing process (3) Residue detection
Heterogeneity caused by nutrient gradients in 3D culture	(1) Optimization of bioreactors (2) Application of cell scaffold
The risk of immune rejection	Cell-free exosome replacement therapy

At present, there are already many proven genetically modified viruses (Lentivirus and AAV) or non-viral (CRISPR/Cas9, transposon and RNA) genetic modification systems. These strategies could introduce exogenous genes either in an integrated or non-integrated form. Generally, we consider that the non-integrated form is safer, but in practical applications some integration strategies have already been applied to the approved cell therapy products such as CASGEVY® and Puzolcabtagene Autoleucel®. Particularly, the targeted insertion mediated by CRISPR/Cas9 avoided the risks associated with random insertion. The genotoxic risk of genetic modification could be comprehensive assessment by integration site detection (Lemmens et al., 2024), missed-target detection (Lei et al., 2023) and clone formation assay (Bastone et al., 2025) during quality control process, which could minimize the risk of genotoxic risk to the greatest extent. However, the long-term safety and reproductive toxicity of transgenic cell therapy still need to be verified through clinical trials.

As such, preconditioning strategies including small molecule treatment provide a feasible workaround to regulate target genes and pathways. Nevertheless, small molecules used for pre-treatment need to be cautiously selected. We need to conduct a thorough assessment of the toxicity of small molecules on MSCs and on the human body. The small molecules such as (Atorvastatin, Melatonin, Rapamycin and so on) which have been approved as medicine have relatively higher safety profiles. Most importantly, the washing process before freezing the MSC preparation can significantly reduce the residual amount of small molecule drugs in the final product. The level of drug residues is also one of the important indicators for QC. Apart from these biochemical adjustments, the optimization of the cultivation process such as 3D culture can also regulate the target gene to a certain extent.

However, during the 3D culture, nutrient gradient may cause heterogeneity. The heterogeneity could be double-edged sword. It causes the uniformity of the product to deteriorate, but also can be designed to guide MSCs to undergo orderly lineage differentiation in 3D space, thereby constructing complex tissues or organoids with functional compartments (Samal et al., 2021). The optimization of bioreactors can enhance the convection and diffusion of nutrients, and can partially alleviate the severe nutrient gradients in static cultures (Sart and Agathos, 2016). Besides, the application of cell scaffold enabled cells to form more uniform spheres better maintain the expression of stemness associated genes, and promote uniform differentiation (Yin et al., 2024). Moreover, MSCs could be a the highly promising seeds for tissue engineering. A single modification strategy at the cellular level cannot make the fullest use of MSCs. Genetic editing grants MSC enhanced capabilities, including cytokine secretion, anti-apoptosis and chemotaxis. 3D dynamic culture not only can enhance the homogeneity of MSCs, but also provides a mechanical microenvironment. Besides, smart responsive scaffolds make the culture process become programmable. Combination of these strategies expected to be possible to directly “print” artificial organs with complex functions *in vitro* for tissue regeneration (Mohammadi et al., 2025).

Although several strategies have been developed to resolve these challenges, the risks of MSC therapy remain inevitable. Cell-free exosome therapy is therefore a promising direction. MSC-derived extracellular vesicles (MSC-EVs) have emerged as a superior acellular alternative with lower immunogenicity, decreased tumorigenic potential, and better storability than intact MSCs (Aguiar Koga et al., 2022; Chattopadhyay et al., 2025). Nevertheless, their clinical translation is hindered by significant batch-to-batch functional variability, even under current good manufacturing practice (GMP) standards (Zhang F. et al., 2024). As demonstrated by Tertel et al., the immunomodulatory capacity of iPSC-MSC-derived EVs differs markedly across independent production batches (Tertel et al., 2023). Despite the current GMP standards, the deficiency of suitable quality-control methods and the difficulties in large-scale preparation largely restrict the development of therapeutic MSC-EV products (Welsh et al., 2024). Challenges such as standardization of isolation protocols, establishment of quality control criteria, and scalability of production remain unresolved (Li S. et al., 2025).

Engineering strategies offer some solutions to overcome the limitations of MSC-EV application. Preconditioning parent MSCs with hypoxia, cytokines, or 3D culture can enhance EV yield and therapeutic efficacy (Song Y. et al., 2025; Yoo et al., 2025). For instance, 3D culture of AD-MSCs significantly increases EV production and improves osteoarthritis treatment outcomes when combined with injectable hydrogels (Fu et al., 2025). Furthermore, scaffold-mediated controlled release addresses the short half-life of free EVs. A 3D-printed hydrogel scaffold loaded with bone MSC-EVs sustained EV release for 1 month, reducing early inflammation and enhancing bone formation (Li M. et al., 2025). These findings suggest that the preconditioning and scaffold strategies discussed earlier for intact MSCs can be effectively translated to EV-based platforms, creating a unified engineering framework for safer regenerative therapies.

In summary, different MSC enhancement strategies each have their respective limitations, covering genetic safety, residual small molecules, nutrient distribution and quality control. The solutions discussed above serve as a practical guide for risk control, and will greatly promote the clinical transformation of next-generation MSC therapies with superior safety and therapeutic performance.

## 5 Conclusion

MSCs exert therapeutic effects through mechanisms like paracrine signaling and multidirectional differentiation. These effects include anti-apoptosis, homing, immunomodulation, angiogenesis, and so on. Owing to this broad therapeutic potential, MSCs have emerged as ideal candidate seed cells for cell therapy. However, the further application of MSCs is limited by their low homing efficiency and poor survival rate in the harsh microenvironment of the injury site. To circumvent these constraints, a variety of engineering strategies have been explored. This review has systematically examined the strategies, including genetic modification, hypoxia preconditioning, cytokine or compound pretreatment, and combination therapy with scaffolds. While these enhancement approaches are varied, they share common mechanistic pathways. For instance, most modification strategies improve MSC survival, homing, paracrine activity, and immune regulation by activating the PI3K/Akt signaling pathway. The selection of optimal strategies must be fundamentally guided by specific clinical requirements. Orthopedic applications, for example, prioritize structural integration and mechanical support through scaffold-based systems. Whereas immune-related disorders may benefit more from genetic engineering approaches that amplify MSC anti-inflammatory and immunoregulatory capacities. Enhanced MSCs may become a new focus of regenerative medicine, immunomodulation, and cell-based therapy.
