# Supplementary material for: Advances in enhanced mesenchymal stem cell technologies: innovations and therapeutic applications
Source: Front Cell Dev Biol. 2026 Jul 20;14:1868610. doi: 10.3389/fcell.2026.1868610 (PMC13429765; doi:10.3389/fcell.2026.1868610)
Supplement: Supplementary file 1 [file Table1.docx]

Supplementary Material

# Supplementary Tables

**Supplementary Table 1** Enhanced scaffolds for MSC regulation.

| **Scaffold types** | **MSCs Type** | **Enhanced Effects** | **Indications** | **References** |
| --- | --- | --- | --- | --- |
| Chitosan membranes | AD-MSCs | Adhesion, homing, anti-inflammatory, differentiation | — | (Yeh et al., 2014) |
| Polycaprolactone/chitosan blend membrane | BMSCs | Invasion, proliferation, promoted cell viability |  | (Das et al., 2019) |
| 3D printed scaffolds |  | Chondrogenic and osteogenic differentiation |  | (Prasopthum et al., 2019) |
| 3D printed gelatin/Hydroxyapatite scaffolds | UCB-MSCs | Proliferation, chondrogenic differentiation | Cartilage defect | (Huang et al., 2021) |
| Osteoconductive scaffolds | AD-MSCs | Proliferation, osteogenic differentiation | Critical size defect | (Kuttappan et al., 2018) |
| Gelatin/nano-hydroxyapatite scaffolds | MSCs | Homing, osteogenic differentiation | Radial bone defects | (Kamali et al., 2019) |
| 3D hydrogel scaffolds | BMSCs | Antioxidant-immunomodulatory, osteogenic differentiation | Fracture | (Qiu et al., 2024) |
| Collagen hydrogel scaffolds |  | Chondrogenic differentiation | Cartilage defect | (Zhou et al., 2019) |
| 3D graphene oxide (GO) incorporated hydrogel | BMSCs | Survival, chondrogenic differentiation | Damaged articular cartilage | (Shen et al., 2020) |
| Kidney ECM hydrogel | AD-MSCs | Proliferation, homing, epithelial differentiation | Renal I/R injury | (Zhou et al., 2020a) |
| Wharton's jelly-derived ECM | WJ-MSC | Adhesion, infiltration, homing, osteogenic differentiation | Fracture | (Beiki et al., 2018) |
| Hierarchically assembled nanofiber scaffolds | BMSCs | Chondrocyte and osteoblast differentiation | Bone defects | (Pan et al., 2024) |
| PLGA nanofiber scaffolds | AD-MSCs | Osteogenic differentiation | Fracture and osteoporosis | (Qi et al., 2021) |
| Poly-L-lactic acid nano fibrous scaffolds | AD-MSCs | Adhesion, proliferation, osteogenic differentiation | Fracture | (Birhanu et al., 2018) |
| Chitosan extruded nanofiber scaffolds |  | Re-epithelialization, angiogenesis, anti-inflammatory | Diabetic ulcer | (Abdollahi et al., 2024) |
| Fibrous scaffolds |  | Paracrine | Skin wound | (Su et al., 2017) |
| Collagen/Tussah silk fibroin scaffolds | BMSCs | Adhesion, viability, differentiation | Skin wound | (Cui et al., 2020) |
| Nano-hydroxyapatite/chitosan scaffolds |  | Adhesion | Bone defect | (Zhang et al., 2018) |
| Chitosan/agarose/gelatin scaffolds |  | Homing, osteogenic differentiation | Fracture | (Wang et al., 2018b) |
| Composite porous poly(dl-lactide-co-glycolide) scaffolds | AD-MSCs | Adhesion, infiltration, proliferation and osteo-differentiation | Fracture | (Casagrande et al., 2018) |
| Polycaprolactone/GO nanocomposite scaffolds | MSCs | Cell activity, osteogenic differentiation | — | (Rostami et al., 2020) |
| Polyurethane/clay nanoplates | AD-MSCs | Proliferation, osteogenic differentiation |  | (Norouz et al., 2019) |
| Hetero-spheroids with quercetin |  | Paracrine, anti-apoptosis, anti-inflammatory | Colitis | (Regmi et al., 2021) |
| Gelatin microcarriers | UC-MSCs | Anti-inflammatory, reduced pathological vascularization (Rap1) | Tendinopathy | (Li et al., 2024a) |
| GMs |  | Paracrine, antioxidation | ALF | (Song et al., 2025a) |
|  | AD-MSCs | Paracrine, upregulated stemness genes | Skin wound | (Zeng et al., 2015) |
| Polystyrene material | BMSCs | Immunomodulatory function | Sterile inflammation | (Kaur et al., 2024) |
| Porous arginine-glycine-aspartic acid (RGD)-modified alginate microcarriers | UC-MSCs | Adhesion, survival, immunoregulation (cytokine-receptor interaction/IL-17) | Dry eye disease | (Chen et al., 2025) |
| Crosslinked polystyrene | UCB-MSCs | Survival, proliferation | — | (Naseri Mobaraki et al., 2022) |
| Micronized amniotic membrane | UC-MSCs | Survival, neovascularization | Burn wound healing | (Zhou et al., 2023) |
| Polycaprolactone microcarriers | BMSCs | Proliferation, osteogenic differentiation | Ectopic mineralization | (Shekaran et al., 2016) |
| Poly (ethylene glycol)-based hydrogel microcarriers |  | Paracrine, angiogenesis (*MAPK*) | — | (Doron et al., 2023) |
